# The Temporal Dynamics of Early Visual Cortex Involvement in Behavioral Priming

**DOI:** 10.1371/journal.pone.0048808

**Published:** 2012-11-14

**Authors:** Christianne Jacobs, Tom A. de Graaf, Rainer Goebel, Alexander T. Sack

**Affiliations:** 1 Department of Cognitive Neuroscience, Faculty of Psychology and Neuroscience, Maastricht University, Maastricht, The Netherlands; 2 Maastricht Brain Imaging Center, M-BIC, Maastricht, The Netherlands; Radboud University Nijmegen, The Netherlands

## Abstract

Transcranial magnetic stimulation (TMS) allows for non-invasive interference with ongoing neural processing. Applied in a chronometric design over early visual cortex (EVC), TMS has proved valuable in indicating at which particular time point EVC must remain unperturbed for (conscious) vision to be established. In the current study, we set out to examine the effect of EVC TMS across a broad range of time points, both before (pre-stimulus) and after (post-stimulus) the onset of symbolic visual stimuli. Behavioral priming studies have shown that the behavioral impact of a visual stimulus can be independent from its conscious perception, suggesting two independent neural signatures. To assess whether TMS-induced suppression of visual awareness can be dissociated from behavioral priming in the temporal domain, we thus implemented three different measures of visual processing, namely performance on a standard visual discrimination task, a subjective rating of stimulus visibility, and a visual priming task. To control for non-neural TMS effects, we performed electrooculographical recordings, placebo TMS (sham), and control site TMS (vertex). Our results suggest that, when considering the appropriate control data, the temporal pattern of EVC TMS disruption on visual discrimination, subjective awareness and behavioral priming are not dissociable. Instead, TMS to EVC disrupts visual perception holistically, both when applied before and after the onset of a visual stimulus. The current findings are discussed in light of their implications on models of visual awareness and (subliminal) priming.

## Introduction

Since the pioneering work of Amassian in 1989 [1], many researchers have reverted to chronometric Transcranial Magnetic Stimulation (TMS) in order to directly test the stimulus-locked functional relevance of early visual cortex (EVC; i.e. V1, V2, V3) for visual perception over time. This elegant experimental approach allows for the spatially and temporally specific interference with regular neural processing in healthy human individuals, and all of these studies robustly showed that visual discrimination is impaired when a single TMS pulse is delivered to EVC approximately 90 ms after the onset of the visual stimulus [1], [2], [3], [4], [5], [6], [7], [8], [9], [10], [11], [12]. In other words, visual discrimination relies on intact EVC activity at ∼90 ms after the visual stimulus is presented.

Later studies included a direct, subjective measure of conscious perception in the form of self-reported awareness [12], [13], [14], [15], [16], [17], and showed that EVC TMS similarly affects subjective visual awareness and discrimination performance [13], [15], [16], [17]. The subjective and objective measures of visual awareness in the current dataset further support this similarity in temporal profile, as was previously published in [18]. Thus, visual processing in EVC ∼90 ms after the presentation of a visual stimulus can be said to contribute to both visual discrimination and constitution of stimulus awareness, as reflected by both objective and subjective measures of visual perception. In spite of this, there is concrete evidence that different neuronal processes underlie different forms of visual processing.

Subliminal perception refers to perception of a stimulus without accompanying conscious awareness. The neuropsychological phenomenon of blindsight is an example of subliminal perception [19], as blindsight patients notoriously report not to experience vision in a part of their visual field, although they score above chance when asked to judge visual stimuli presented in their blind spot. Under experimental conditions it is possible to evaluate subliminal perception in healthy observers as well [20]. The existence of subliminal perception raises the intriguing question to what extent the content of subliminal stimuli can steer later behavior, even if the perceiver does not report any conscious awareness of the stimulus. This issue has driven much psychophysical research on subliminal perception. Masked priming studies showed that a visual stimulus can indeed modulate subsequent behavior in the absence of visual awareness, and that this can occur at different levels of the visual hierarchy, from low-level color and form priming [21], [22], [23] all the way up to semantic priming [24], [25], [26], [27], [28]. The dissociation between awareness and behavioral influence, as apparent in all these psychophysical studies, triggered the search for the independent neural mechanisms underlying visual awareness and behavioral priming, which many suspect to involve early visual cortex [10], [11], [29], [30].

Our group recently conducted a chronometric TMS study to investigate whether the visual suppression caused by disrupting EVC at the classical post-stimulus time window leaves the behavioral impact of this stimulus, which had previously been shown not to require visual awareness [23], on a second stimulus unaffected. The study revealed that both masking and priming functions break down when EVC TMS is applied 80–100 ms after the onset of the first visual stimulus in a combined masking-priming experiment [11], suggesting that both conscious and unconscious visual processing, as reflected by discrimination performance and priming, rely on intact processing in EVC around ∼90 ms post-stimulus onset. This conclusion was further corroborated by a study which examined priming of metacontrast masked stimuli after chronometric EVC TMS and also reported a reduction in priming 30–90 ms post-prime [31]. Even though the latter study had a slightly broader temporal scope (30–180 ms post-stimulus), these findings do not rule out the possibility that a dissociation between visual awareness and behavioral priming might still exist in the temporal domain at other critical time periods of stimulus-related activity within EVC. This becomes particularly important when considering the recently established additional time windows of relevant EVC activity for visual processing, including time points later than 180 ms post-stimulus, and even time points prior to visual stimulus onset.

Concretely, in addition to the long established post-stimulus TMS time window of ∼90 ms for visual suppression, EVC TMS has been shown to interfere with visual discrimination performance at an additional time point around 200 ms post-stimulus [3], [16], [32]. Conscious perception has been repeatedly linked to feedback processing [33], [34], [35], [36], [37], and the late effective TMS time window has been suggested by some to reflect recurrent processing in EVC [3], [16], [32]. If activity in EVC at this stage dissociates between conscious and unconscious perception, priming, a process that does not require visual awareness, should remain unaffected. To our knowledge, so far none of the studies employing chronometric EVC TMS in a (masked) priming paradigm were able to address this idea, generally not systematically testing such late TMS time windows for different measures of visual processing.

Another time frame in which EVC TMS was found to disrupt normal visual perception, is a remarkable one, because it precedes the onset of the visual stimulus [2], [4], [6], [9], [17], [18]. The neural mechanisms underlying such a potential pre-stimulus EVC TMS suppression effect have to be fundamentally different from those underlying post-stimulus TMS. The post-stimulus TMS suppression effect has been claimed to reflect a decreased signal-to-noise ratio exactly at that critical time period when the stimulus-related signal is being processed in EVC [38]. However, at any pre-stimulus TMS time window affecting visual perception, the stimulus-related signal has not even reached EVC. Therefore, some of the few studies that revealed a pre-stimulus TMS visual suppression effect attributed this effect to TMS-induced eye blinking [4], [39], [40]. Recently, however, we presented first empirical evidence that the suppressive influence of pre-stimulus TMS on visual perception remained present even after controlling for eye blinks or other non-specific TMS effects, suggesting that the pre-stimulus TMS effect on visual perception may after all be of neural origin [18]. This raises the question whether the pattern of behavioral consequences of pre-stimulus TMS is different to the pattern of behavioral consequences of post-stimulus TMS, and whether two neural processes underlying visual awareness and behavioral priming are dissociable in the pre-stimulus temporal domain?

The current study aimed to address these questions by testing whether EVC TMS at any pre- or post-stimulus time window selectively hinders the constitution of visual awareness while, e.g., leaving a potential subliminal behavioral priming effect intact, or whether it affects visual processing holistically, including objective recognition, subjective awareness, and behavioral priming. To this end, we went beyond previous work by employing a rigorous, systematic experimental paradigm covering a wide range of TMS time windows, a wide range of behavioral measures, and multiple forms of TMS control. Thus, we measured both task performance and self-reported visual awareness in the context of a visual discrimination task, and we measured the behavioral impact of identical visual stimuli in a symbolic behavioral priming task. On each trial a single TMS pulse was delivered over EVC at one of 20 different time points time-locked to the first visual stimulus and ranging from −80 to 300 ms in steps of 20 ms. This extensive chronometric TMS design ensured to test several time windows pre- as well as post-visual-stimulus onset. In addition, by broadening our temporal scope further into the post-stimulus domain than previous work, we could explore whether EVC selectively affects visual awareness at any time point beyond the classical TMS-induced masking time window of 80–100 ms.

Throughout the experimental sessions, electrooculographical (EoG) data were recorded to control for eye blinks. Additionally, in a separate sham TMS session, EVC was stimulated with a placebo TMS coil to account for the influence of TMS-related auditory stimulation. As a final control for non-specific TMS effects, a non-relevant site (vertex) was stimulated with genuine TMS using the identical chronometric design as applied over EVC.

## Materials and Methods

### Ethics Statement

The study was approved by the medical-ethical committee of the University Medical Center, Maastricht, the Netherlands. Prior to the experiment, all participants were requested to fill out a medical questionnaire, which was screened and approved by a medical supervisor. At the start of each experimental session, participants filled out an additional questionnaire to check whether current circumstances allowed TMS application. All participants gave written informed consent at the start of each session, and were compensated financially for their participation.

### Participants

18 healthy participants (6 males, mean age 23.6 y, range 19–32) with normal or corrected-to-normal vision participated in this study, including one of the authors (CJ). 10 Participants received TMS at the experimental site EVC, whereas the other 8 received TMS at a control site (vertex).

Three participants were excluded from further analysis (two in main experiment and one in the vertex control experiment), when their baseline accuracy in the recognition task proved to be below 75% correct responses.

### Stimuli

Two schematic drawings of horizontal arrows on a white background served as stimuli. They were presented serially on each trial of the recognition as well as the priming task, and were located in the horizontal center of the lower visual field, 0.8° visual angle below fixation. The first stimulus (S1) was a small (0.8° by 1.86°) arrow presented for 16.7 ms. It was followed by a larger arrow stimulus (S2) which consisted of black outer contours only, and was on screen for 66.7 ms. Both arrow stimuli pointed in either leftward or rightward direction and their relative stimulus-onset asynchrony was fixed at 83.3 ms. Stimulus presentation and recording of behavioral responses was accomplished through the Presentation software package (Neurobehavioral Systems, Inc., Albany, CA).

Stimuli were shown on a 17″ TFT, Samsung SyncMaster 931 DF computer monitor with a refresh rate of 60 Hz. Calibration measurements using a photodiode on the monitor and concurrent measurement of the external TMS triggering signal leaving the parallel port of the stimulus pc showed a constant, stable and reliable offset of 2 ms for all stimulus-TMS SOAs. When we discuss SOAs in the remainder of the paper, we refer to the SOAs as requested of our presentation software.

### Experiment

A within-subject design was employed in which participants performed two different two alternative forced-choice (2AFC) visual discrimination tasks. The study asked for an extensive dataset, because of the large amount of TMS time windows under investigation here. To prevent participant fatigue, data collection was spread over four two-hour sessions per participant. In two out of four sessions, the recognition task was performed; in the other two, the priming task was performed. Session order was counterbalanced across participants. All stimulus and TMS parameters were identical for both tasks, only task instruction differed. At the start of each session, participants were comfortably seated in front of the monitor, and their heads were stabilized in a chin-rest.

In the *recognition task*, participants covertly paid attention to the direction of the first symbolic arrow, S1. They were instructed to retain fixation throughout the experiment and to be as accurate as possible in their responses. To measure their subjective visual awareness, they were asked to indicate via button press whether they consciously perceived S1 or not. All participants were instructed to respond positively to this question when their percept entailed some informational content, even if they would still be unsure on stimulus direction. By their second key press, participants indicated which direction, i.e. left or right, they believed S1 pointed to. The task was set up as a 2AFC, so it was not possible for participants to continue the experiment unless two responses were given on each trial. Participants were explicitly informed that the two responses were independent, i.e. indicating that S1 was consciously perceived did not mean that a correct answer on its direction was required.

Participants were trained on the task until they reached an average accuracy of at least 80% correct responses on three consecutive blocks of 20 trials with a maximum of 15 blocks. If the accuracy threshold was not reached within these 15 blocks, the participant was not included in the study. At single trial level, performance was indicated by the fixation cross, which would turn green after correct responses and red after incorrect responses. During breaks, summary feedback was given to the participants about their performance on the previous block.

**Figure 1:**
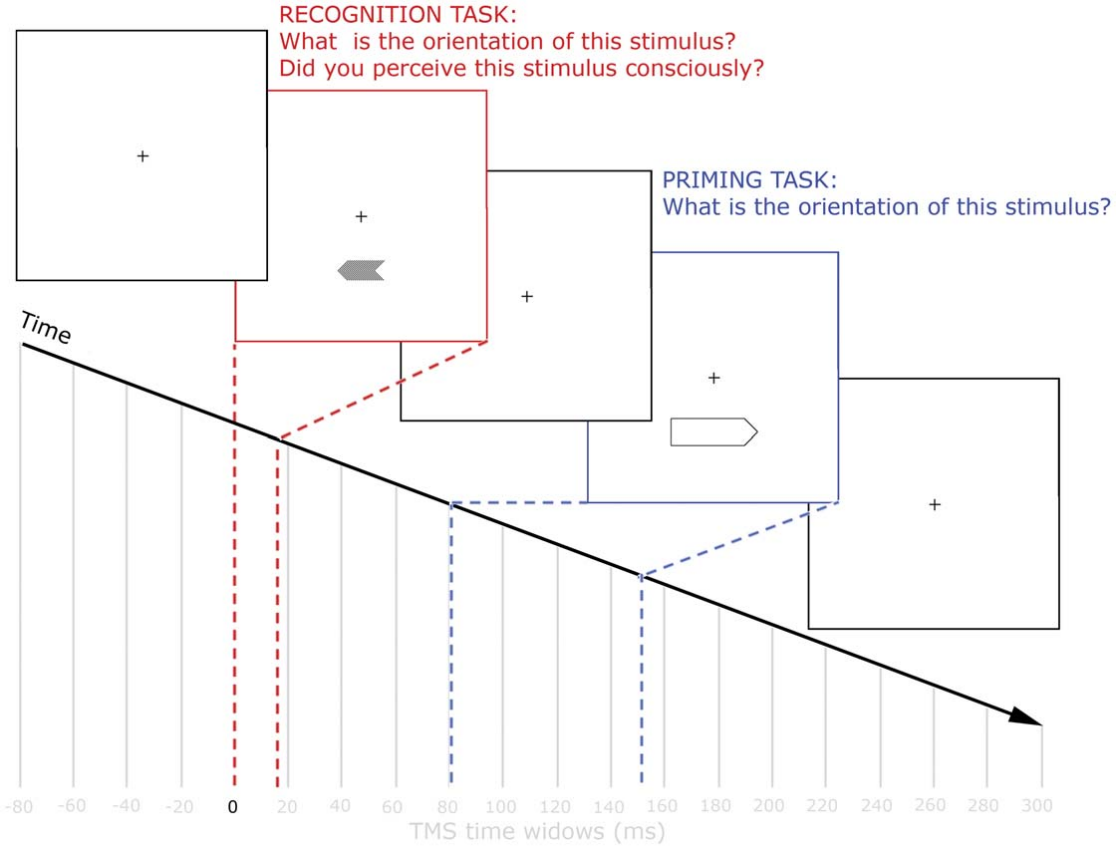
Trial time line. A horizontal black arrow (S1) was presented below fixation for 16.7 ms, followed by a larger horizontal arrow (S2) for 66.7 ms with an SOA of 83.3 ms. During each trial a single TMS pulse was delivered over EVC at any of 20 different time points time-locked to S1 (range from −80 to 300 ms in steps of 20 ms). In the recognition task, participants were required to indicate via button press whether they had perceived the *first* arrow consciously or not. By a second button press they indicated whether they thought the *first* arrow pointed leftward or rightward. In the priming task, participants were required to indicate via button press whether they thought the *second* arrow pointed leftward or rightward.

Each session included a No TMS baseline measurement. This measurement consisted of 20 trials during which no feedback was given and no TMS pulses were delivered. Participants then completed 400 TMS trials, divided over 20 blocks. On each of these trials a single TMS pulse was randomly delivered at one of 20 TMS time windows ranging from −80 to 300 ms post S1-onset in 20 ms steps (see Figure 1). Inter-trial-interval was jittered and had an average duration of 5000 ms. In each session 20 trials per time window were collected leading to a total number of 40 trials per time window.

In the behavioral *priming task* participants responded to the direction of S2, via button press and as quickly as possible. In this task, the main goal was to measure the behavioral priming effect of S1 on the reaction times (RTs) to S2. RTs to S2 were expected to be shorter in case S1 pointed in the identical direction (i.e. congruent trial) than when it pointed in opposite direction (i.e. incongruent trial). No measure of subjective awareness was implemented here since visual awareness of S2 was not deemed relevant to the research question. No training was offered to participants, since the priming task was fairly easy and the dependent variable in this task does not depend on any prior training.

### TMS protocol

Phosphene localization was used to determine coil position. The initial position of the TMS coil was approximately 1 cm above the inion. While the participants were fixating, they received single pulses of TMS. Participants reported whether they perceived TMS-induced phosphenes, and if so, where these were located within their visual field. Coil position was then systematically varied until the induced phosphenes overlapped with the visual field location of the experimental stimuli. If complete overlap could not be achieved, the coil position was chosen at which induced phosphenes were closest to the desired location. TMS applied over stimulation sites based on phosphene localization has been shown to most probably target V2/V3 [41], [42].

During each trial a single biphasic TMS pulse was administered at any of 20 TMS time windows. TMS time windows were all time-locked to S1 ranging from −80 to 300 ms post-S1 onset in 20 ms steps. The stimulation intensity was fixed across participants and set to 70% of maximal stimulator output (Medtronic Functional Diagnostics A/S, Skovlunde, Denmark; maximum stimulator output = 1.9 T). Phosphene thresholds in a subset of our participants were 51% maximum stimulator output on average. All pulses were administered with a figure-of-eight coil (MC-B70, the inner and outer radii of the two coil loops are 1.2 and 5.4 cm, respectively). The TMS coil was positioned horizontally, with the coil handle pointing rightwards.

### Control measures for non-neural TMS effects

TMS is known to also induce non-neural side-effects due to the confounding acoustical and sensorimotor stimulation inherent to any TMS protocol. Therefore, we included a number of complementary control measures [43] to account for such possible non-neural effects of TMS on our visual awareness and behavioral priming measures.

First, electrooculography (EoG) data were recorded to collect information on the participants' vertical eye movements. Trials containing an eye blink in the time interval −200 to 100 ms relative to S1 onset were labeled ‘blink’ trials, and excluded from the subsequent analyses on time-specific TMS-induced masking or priming (for a detailed description and comparison of the data before and after eye blinks removal and their putative role in pre-stimulus masking, please consult [18]).

Second, to control for the clicking sound accompanying TMS pulses, data of the experimental sessions were compared to data acquired in a sham TMS session using a specific TMS placebo coil (MC-P-B70 Placebo) with 6 participants of the original sample. The sham TMS pulse was randomly delivered at any of the original pre-stimulus time windows (i.e. −80, −60, −40, −20, 0 ms). The measured time windows were limited to the pre-stimulus domain, because it would be too strenuous on the participants to undergo another four sessions of data acquisition, especially since we expected the auditory click to act as a warning signal and therefore have its biggest alerting influence when it precedes the visual stimulus [44].

Third, to also control for the aversive sensations on the head resulting from magnetic stimulation of the scalp that the placebo TMS coil does not mimic, we decided to re-run the entire experiment with a different group of subjects, this time stimulating vertex as control site with real TMS instead of EVC. Vertex was defined as Cz in the 10–20 electrode positioning system.

For direct comparison to the EVC experimental data, acquiring the vertex data in the original participant sample would have been optimal. However, because we failed to find all original participants able and willing to invest in the study for another four sessions, and since we were cautious for a lack of motivation in participants when having to perform too many task repetitions, we decided to acquire the vertex data in a new participant sample as a between-subject factor.

### Analyses

On the basis of the EoG data, trials were classified as ‘blink’ or ‘no blink’ trials. Trials were considered blink trials if an eye blink was detected anytime between −200 ms prior and 100 ms post S1-onset (for details on the analysis of the EoG data, see [45]). After analyses of the complete EVC TMS dataset, the eye blink trials were removed, and the analyses were repeated for residual data.

In the recognition task, we measured both subjective visual awareness and recognition accuracy. Subjective visual awareness was indicated by participants on a two-point scale, corresponding to whether they regarded S1 as ‘seen’ or ‘unseen’. The percentage of ‘seen’ responses was then calculated per condition, i.e. per TMS time window. In a similar fashion, the percentage of correctly identified trials per time window served as our measure of accuracy. We calculated these values for each subject individually and performed one-way repeated-measures analysis-of-variance (RM-ANOVA) of the factor Time Window separately on both dependent variables. Post-hoc analyses comprised of comparisons of all 20 experimental levels of the factor Time Window to the baseline condition No TMS. The alpha values for these pairwise comparisons were least-square difference (LSD) corrected. Note that the data of the recognition task have been reported before, in [18]. Analyses then focused on the comparison of the visual suppression effect before and after removal of ‘blink’ trials. Since we here compare the pattern of results from the recognition task with that of the priming task, we show these data here again.

In the behavioral priming task, reaction times (RTs) were taken as a dependent variable. Outlier analysis consisted of excluding those trials on which RTs exceeded the 1.5xIQR criterion per participant. Only RTs of correct trials were analyzed. Average RTs were calculated for each TMS time window and for congruent (Con) and incongruent (INCon) trials separately. The average RTs on congruent trials were subtracted from the average RTs on incongruent trials resulting in a measure of behavioral priming which we termed the priming effect (PE). PE scores were calculated for each TMS time window in each individual. On the priming data a two-way RM-ANOVA was conducted with factors Time Window (21 levels; 20 TMS time windows and No TMS baseline) and Congruency (2 levels). The factor Congruency was then collapsed into the single measure of the PE and PE data were submitted to a one-way RM-ANOVA with Time Window as the single factor. Again, post-hoc analyses consisted of comparisons of all 20 experimental levels of the factor Time Window to the baseline condition No TMS, and alpha values for these pair wise comparisons were LSD corrected.

The data of the vertex control experiment were analyzed in the same way as described for the experimental data. To compare the vertex and EVC TMS data a two-way mixed ANOVA was performed with Time Window (21 levels) as within-subject factor and Site (2 levels) as between-subjects factor. Significant interactions were further investigated by means of independent samples t-test comparing the group data per time window.

For the recognition part of the sham TMS session, average accuracy and awareness scores per participant and per sham TMS time window were calculated. These two measures served as input for a two-way RM-ANOVA with factor TMS Type (2 levels: Sham versus EVC TMS) and Time Window (21 levels), followed by a one-way RM-ANOVA on the sham data with single factor Time Window. For the priming part of the sham TMS session, PEs were calculated per participant and per sham Time Window (see above). Again, a two-way RM-ANOVA including factors Type and Time Window and a one-way RM-ANOVA on the sham TMS data were then conducted. Post hoc analyses consisted of pair wise comparisons of the 5 sham TMS time windows to No TMS baseline with LSD corrected alphas.

For all ANOVAs conducted, the Huyn-Feldt correction for degrees of freedom was applied, if equal variances could not be assumed.

## Results

### Recognition task

#### Accuracy

**Figure 2:**
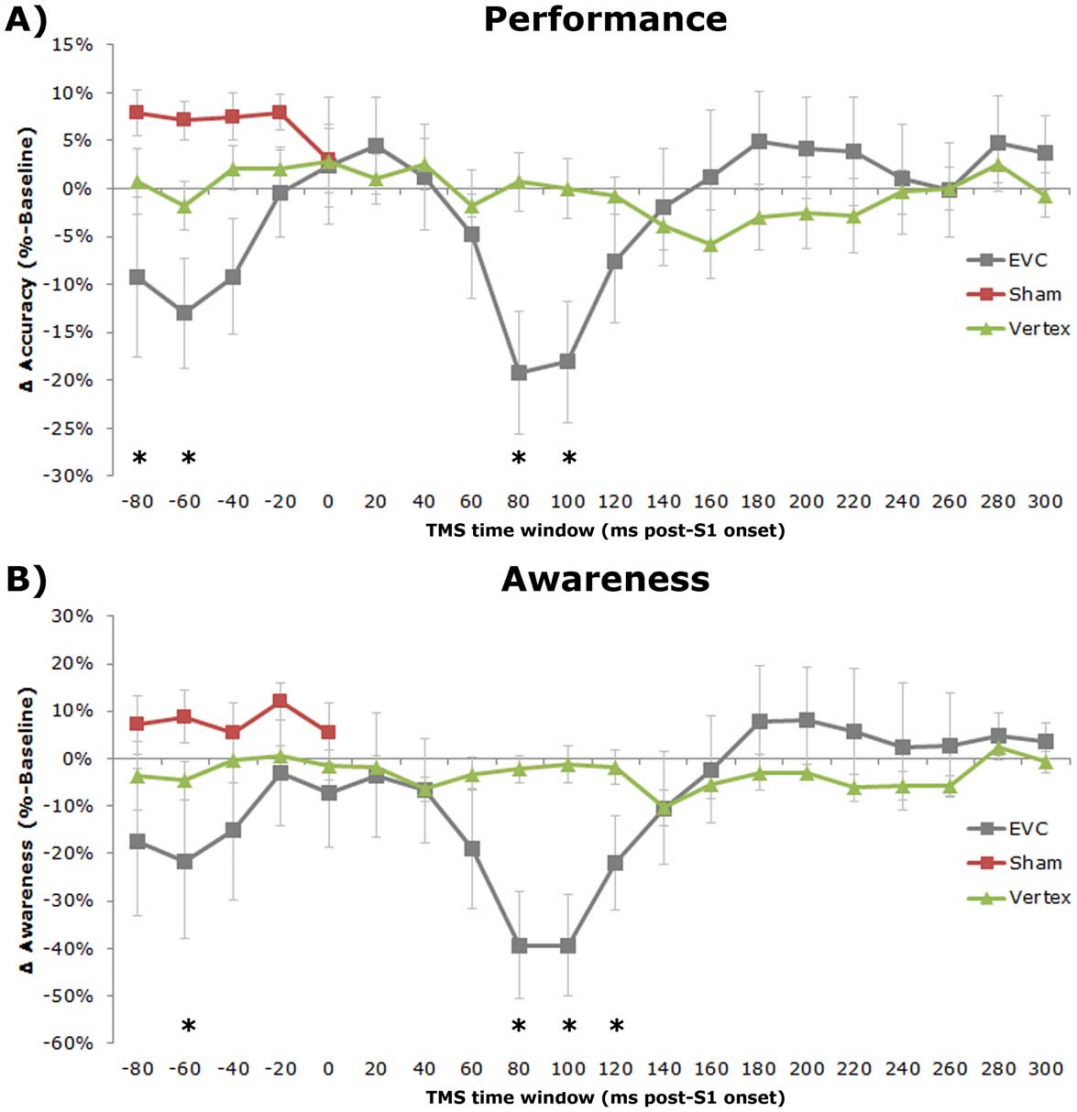
Recognition task data. A) Accuracy relative to baseline (No TMS) for the experimental EVC TMS (grey line), control Sham TMS (red line), and Vertex TMS (green line) datasets, expressed as the percentage of correct responses. Error bars indicate standard errors of the mean. Asterisks indicate EVC TMS time windows in which the accuracies are significantly different from baseline. B) Awareness ratings relative to baseline (No TMS) for the experimental EVC TMS (grey line), control Sham TMS (red line), and Vertex TMS (green line) datasets, expressed as the difference in percentage ‘seen’ stimuli. Error bars indicate standard errors of the mean. Asterisks indicate EVC TMS time windows in which the awareness ratings are significantly different from baseline. This figure represents data already published in [18], and is presented here again in a modified version to allow for a direct comparison with the behavioral priming effects.

The one-way RM-ANOVA showed a main effect of TMS time window on the average percentage correct responses (F(8.244, 49.463) = 4.083, p = .001). Post hoc analysis comparing all TMS time windows to baseline revealed that recognition accuracy dropped significantly compared to No TMS (mean = 91.9% correct) when a TMS pulse was delivered at 80 (mean = 70.0% correct, p = .009) or 100 ms (mean = 71.2% correct, p = .01) after S1 onset, and at −80 (mean = 77.5% correct, p = .042) or −60 ms (mean = 77.5%, p = .023) prior to S1 onset (see Figure 2A).

**Figure 3:**
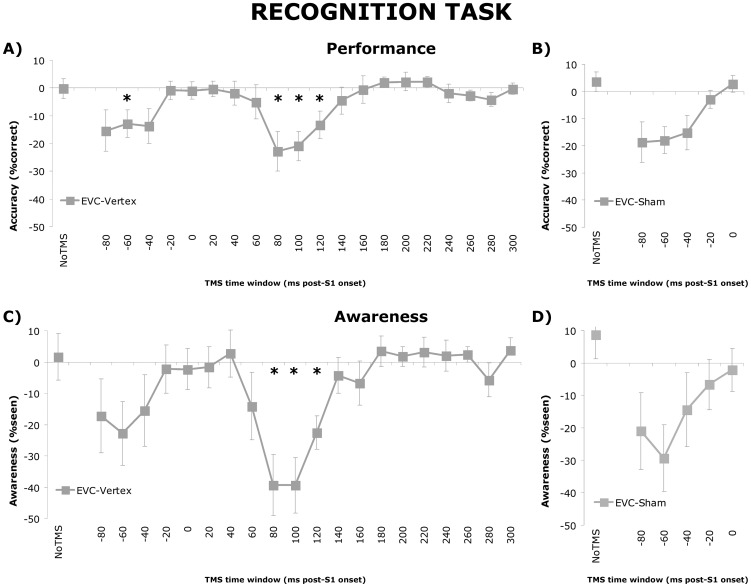
Recognition task data. A) Difference scores between accuracies, defined as the percentage correct responses, in the EVC TMS group versus the average accuracy in the Vertex TMS group per TMS time window. Error bars represent standard errors of the mean (SEM). B) Difference scores between accuracies, defined as the percentage correct responses, in the EVC TMS sessions versus the average accuracy in the Sham TMS session per TMS time window. Error bars represent SEM. C) Difference scores between awareness, defined as the percentage of trials indicated as seen, in the EVC TMS group versus the average awareness in the Vertex TMS group per TMS time window. Error bars represent SEM. D) Difference scores between awareness, defined as the percentage of trials indicated as seen, in the EVC TMS sessions versus the average awareness in the Sham TMS session per TMS time window. Error bars represent SEM. This figure represents data already published in [18], and is presented here again in a modified version to allow for a direct comparison with the behavioral priming effects.

In contrast to the time-specific effects of EVC TMS, sham TMS using a placebo TMS coil did not have a significant effect of Time Window on accuracy scores (F(1.360, 6.798) = 2.009; p = .197). This was confirmed by the significant interaction effect (F(5, 25) = 7.986; p<.01) between TMS Type and Time Window as was found in the conjoined two-way RM-ANOVA on the sham and EVC data (see Figure 3B and [18]).

The two-way mixed ANOVA with Site (EVC vs vertex) as between–subject factor and TMS Time Window as within-subject factor demonstrated a main effect of Time Window (F(4.92, 59.09) = 4.36; p<.01) and a significant interaction effect of TMS Time Window×Site (F(4.92, 59.09) = 4.65; p<.01). Independent samples t–tests comparing EVC to Vertex groups for each time window revealed that accuracy differed between groups when TMS was applied 80 (p = .01), 100 (p = .01) and 120 ms (p = .03) post-stimulus. In the pre-stimulus domain a significant effect of stimulation site was apparent in the −60 ms time window (p = .03). Furthermore, trends were observed −40 (p = .06) and −80 ms (p = .07) pre-stimulus time windows (see Figure 3A).

The one-way RM-ANOVA on the vertex stimulation data in isolation revealed a significant effect of Time Window (F(14.268, 85.607) = 1.906; p = .036), but post hoc comparisons did not show significant changes in accuracy of TMS applied at any tested time window compared to baseline (see Figure 2A).

Together, these results clearly indicate that time-specific pre- and a post-stimulus TMS pulses applied to EVC (and not vertex) lead to reduced visual discrimination ability.

#### Self-reported awareness

As on accuracy, TMS time window had an effect on subjective awareness rating (F(5.166, 30.9995) = 4.525, p = .003). Compared to No TMS (mean = 84.9% seen), awareness was significantly reduced when EVC TMS was applied at −60 ms pre-stimulus (mean = 55.7% seen, p = .043), and 80 (mean = 41.5% seen, p = .006), 100 (mean = 42.7% seen, p = .005), and 120 ms (mean = 58.8% seen, p = .023) post-stimulus time windows (see Figure 2B and [18]).

Both sham TMS (F(5,25) = 2.009; p = .112), as well as vertex TMS (F(5.503, 33.015) = 1.249; p = .308) did not lead to a significant effect of Time Window on self-reported visual awareness (see Figure 2B). Again, the two-way RM-ANOVA revealed a significant interaction effect (F(5,25) = 3.440; p = .017) of Type and Time Window.

As was the case for the accuracy data, the mixed ANOVA with Site (EVC vs vertex) as between–subject factor and TMS Time Window as within-subject factor demonstrated a main effect of Time Window (F(5.03, 60.31) = 3.93; p<.01) and a Time Window×Site interaction effect (F(5.03, 60.31) = 4.23; p<.01). Post-hoc analyses showed that TMS applied at 80 (p = .01), 100 (p<.01) and 120 ms (p = .02) post-stimulus affected the experimental and the control group differently, and a trend towards the same effect was observed for −60 ms TMS (p = .08; see Figure 3C).

### Behavioral priming task

**Figure 4:**
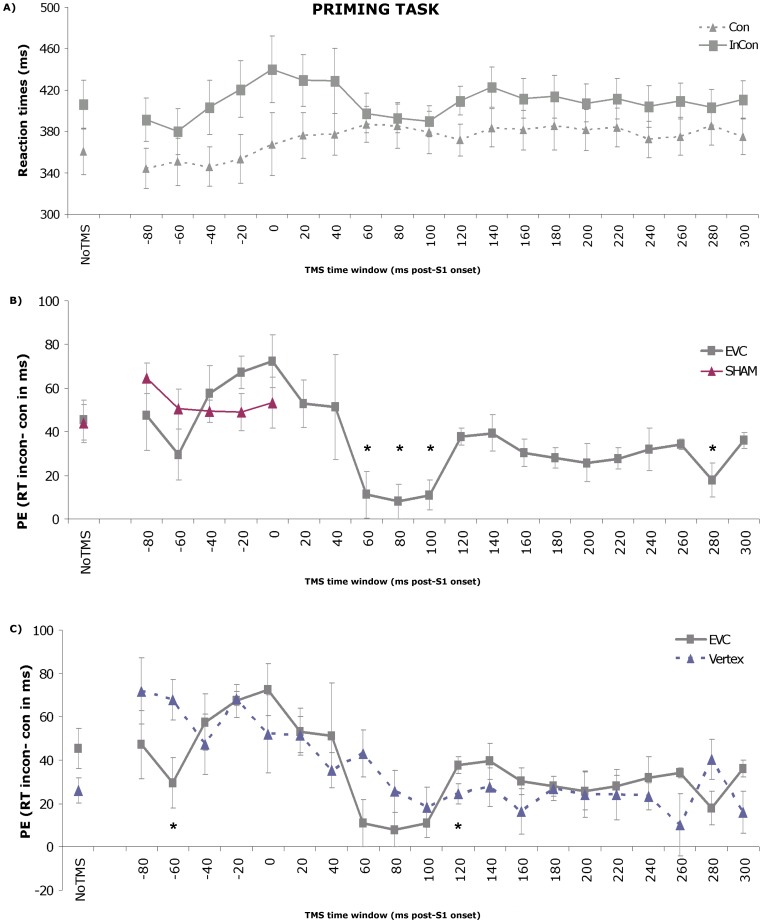
Behavioral priming data. A) Reaction times (RT) to congruent (solid line) and incongruent (dashed line) S2 stimuli per TMS time window. Only experimental (i.e. EVC) data are depicted here. Error bars represent standard errors of the mean (SEM). B) The average priming effect (PE), defined as the reaction times (RTs) in milliseconds on incongruent trials minus the RTs on congruent trials, across TMS time windows is plotted for both the EVC TMS data (grey line) and the Sham TMS data (purple line). Error bars represent SEMs. Significant differences relative to No TMS are indicated with an asterisk. C) The average priming effect (PE), defined as the reaction times (RTs) in milliseconds on incongruent trials minus the RTs on congruent trials, across TMS time windows for both the EVC and Vertex TMS data. Significant differences between the two groups are indicated with an asterisk.

During the preprocessing stages, 6.2% blink trials, 4.5% outliers and 3.8% incorrect responses were removed from the dataset. The two-way RM-ANOVA conducted on the cleaned EVC data revealed a significant main effect of Congruency (F1,7 = 284.43; p<.01), with higher estimated average reaction times for incongruent (mean = 408.9 ms) than for congruent trials (mean = 372.5 ms). Moreover, a significant interaction between the factors Congruency and Time Window (F(5.42, 37.94) = 2.97; p = .02) (see Figure 4A) was revealed.

The subsequent one-way RM-ANOVA on the PE including post-hoc comparisons of the different levels of Time Window was performed to quantify the effect of chronometric TMS on the behavioral impact of the stimulus. Reflecting the dependence of the prime's behavioral impact on timing of the TMS pulse, as was already demonstrated by the interaction effect in the two-way RM-ANOVA, this analysis showed a main effect of Time Window (F(5.42, 37.94) = 2.97; p = .02)

Post-hoc analyses demonstrated that the priming effect decreased significantly compared to No TMS (mean PE = 45.5 ms (Con = 360.7 ms; INCon = 406.2 ms)) when single-pulse TMS was applied 60 ms (mean PE = 11.1 ms (Con = 386.6 ms; INCon = 397.7 ms), p = .039), 80 ms (mean PE = 8.0 ms (Con = 385.3 ms; INCon = 393.3 ms), p = .041), 100 ms (mean PE = 11.0 ms (Con = 379.0 ms; INCon = 390.0 ms), p = .011) and 280 ms (mean PE = 17.9 ms (Con = 385.5 ms; INCon = 403.4 ms), p = .03) post S1-onset. The pre-stimulus time windows, at which TMS negatively affects subjective and/or objective measures of visual perception in the recognition task, did not show any significant decrease in PE in the priming task (time window −80: p = .91; time window −60: p = .33), suggesting at this point that priming can be dissociated from visual awareness by pre-stimulus EVC TMS.

The two-way RM-ANOVA with factors Type and Time Window showed a main effect of Time Window (F(5, 25) = 2.808; p = .038), as well as a significant interaction between both factors (F(5, 25) = 5.459; p = .002). However, the sham TMS data did not reveal an effect of Time Window on priming (F(5, 25) = 1.332, p = .283), as shown in the one-way RM-ANOVA, suggesting that sham TMS did not affect priming in a temporally specific fashion, or in comparison to baseline performance (see Figure 4B).

The two-way mixed ANOVA with Time Window as within-subject factor and Site as between-subject factor showed a significant main effect of Time Window and a trend towards an interaction effect of Time Window×Site (F(8.83, 114.78) = 1.88; p = .063). The independent samples t-tests comparing the EVC and Vertex data per time window revealed a group difference for the 120 ms (p = .05) post-stimulus and −60 (p = .03) pre-stimulus time windows, and trends for the 60 (p = .06), 280 (p = .08) and 300 ms (p = .06) post-stimulus time windows. These data indicate that the trend towards interaction is not solely driven by the different in post-stimulus PE, but also by the smaller pre-stimulus (-60 ms) PE in the EVC versus the Vertex group.

This set of findings now seemed contradictory: the EVC results in themselves did not show any pre-stimulus TMS effect on priming, yet when taking into account the Vertex control data the group analysis suggested a pre-stimulus TMS effect on PE after all. The situation is resolved by considering the vertex data in isolation: the factor Time Window proved to affect behavioral priming (F(14.25, 85.49) = 5.12; p<.01). Post-hoc comparisons revealed an increase in priming compared to baseline (mean PE = 26.0 ms) for the pre-stimulus time period, which reached significance for the −80 (mean PE = 71.9 ms, p = .011), −60 (mean PE = 67.8 ms, p = .001), and −20 ms (mean PE = 68.4 ms, p<.01) time windows. The 20 (mean PE = 48.4 ms, p = .061) and 60 ms (mean PE = 40.6 ms, p = .07) post-stimulus time windows demonstrated a trend in the same direction (see Figure 4). Together, these results indicate that pre-stimulus and early post-stimulus TMS over vertex lead to an enhancement of behavioral priming. As vertex was chosen as an irrelevant control site, we presume that this enhancement is a consequence of non-neural side effects of TMS stimulation (see Figure 4C).

**Figure 5:**
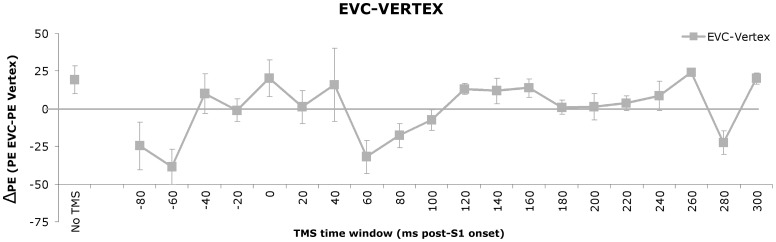
Estimation of the net neural TMS effect. Average priming effect (PE) in the EVC TMS experiment per TMS time window after subtraction of the average PE across participants in the Vertex TMS experiment. Error bars represent standard errors of the mean.

This finding thus suggests an interesting situation: the non-neural effects of TMS at pre-stimulus windows seem to have an enhancing effect on PE (as shown by increased PEs by vertex stimulation). Yet, this enhancing effect is not apparent in the EVC condition, thus likely *counteracted* by the neural suppressive effects of pre-stimulus TMS over occipital cortex. This would suggest that pre-stimulus TMS over EVC indeed suppresses the priming effect (analogous to its suppressive effects on recognition accuracy and visual awareness). To visualize the suggested veridical time course of *neural* TMS effects on PE, we subtracted the EVC results from the Vertex results in Figure 5.

## Discussion

The current study employed an extensive chronometric TMS design including several complementary control measures in order to reveal the temporal pattern of early visual cortex (EVC) involvement in conscious visual perception and its relation to symbolic behavioral priming. To this end, single-pulses of EVC TMS stimulation at 20 time points ranging from −80 to 300 ms post-stimulus onset were applied in steps of 20 ms while participants performed two different two alternative forced choice (2AFC) tasks, i.e. visual discrimination and behavioral priming tasks, assessing objective discrimination, subjective awareness, and behavioral priming of visual stimuli. In order to assess the effects, both neural and non-neural, of chronometric TMS on standard task performance, the experimental data of the 20 tested TMS time windows were compared to a TMS-free baseline condition. After controlling for eye blinks, EVC TMS led to a diminished discrimination performance, when applied 80 and 100 ms post-stimulus onset, and when applied −60 and −80 ms prior to stimulus onset. Self-reported visual awareness was impaired by 80, 100 and 120 ms post-stimulus TMS, and by −60 ms pre-stimulus TMS. Behavioral priming was affected by 60, 80 and 100 ms post-stimulus TMS but at first glance not by pre-stimulus TMS. However, the vertex TMS data (in contrast to the sham TMS data) also showed a time-specific effect on behavioral priming, thus implying that the priming effect is sensitive to the non-neural acoustic and sensorimotor side effects of TMS. Priming was increased by vertex TMS, particularly when the TMS pulse preceded the prime stimulus. Compared to vertex TMS, EVC TMS diminished priming, also in the pre-stimulus domain.

### Post-stimulus effects of EVC TMS on visual perception

Taken together, we here revealed two distinct time periods at which an intact EVC is necessary for the generation of visual awareness. First, TMS successfully masks the visual stimulus when applied at the time windows 80–120 ms post-stimulus onset, a result in line with the vast amount of studies that already established EVC's role in visual processing within this time period [1], [2], [3], [4], [5], [6], [7], [8], [9], [10], [11], [12]. Both objective and subjective measures of visual awareness, i.e. task performance and self-reported awareness, respectively, captured this perceptual deficit, as both dependent variables suffered when the TMS pulse followed the visual stimulus by 80–100 ms. Nevertheless, if the time courses of subjective awareness and objective recognition performance are similar, there could still be a difference in the relative effect sizes [46], [47]. (In fact, for some effective TMS time windows, accuracy scores for ‘unseen’ trials exceed chance level. See [18] for detailed description and discussion.) Behavioral priming is also affected by EVC TMS when applied 60, 80, 100 ms post-stimulus onset, indicating decreased impact of the visual stimulus on subsequent behavior, and suggesting that at least for the post-stimulus temporal domain, behavioral priming and visual awareness are not dissociative within EVC. This finding is in excellent accordance with our and other's previous findings on the interdependence of awareness and priming at post-stimulus EVC TMS time windows [11], [31].

In addition to our previous findings, our design now enabled us to broaden our temporal scope further into the post-stimulus domain. The later TMS time window of visual suppression, potentially reflecting feedback from higher (extrastriate) cortical areas was not replicated in this study. Therefore, the hypothesized dissociation of priming and visual awareness was not found for post-stimulus timings later than the classical masking time window. We did reveal an unexpected effect on priming at 280 ms post-stimulus onset. However, we believe that this very late time window of 280 ms reflects an effect TMS had on the perception of S2, the target stimulus in the priming task, because it does not reveal itself in the recognition task at all.

From psychophysical studies, we know the concept of subliminal priming, i.e. a stimulus that does not cross the threshold for conscious awareness can still influence subsequent behavior, which can be instantiated through visual masking [23], [48], [49], [50]. In this light, it is striking that no such dissociation appears in our data. There are theoretical reasons to expect the two processes to be dissociable in the temporal domain.

First, based on the idea that the recurrent activity looping between striate and extrastriate areas is a prerequisite for visual awareness, at least two post-stimulus time windows would be predicted to show visual awareness impairment; an early one that reflects the interruption of the feedforward stream and a later one that reflects the prevention of feedback. Behavioral priming also depends on an intact geniculo-striate pathway, as we have demonstrated in an earlier study [11]. But, because priming does not require a conscious percept of the prime stimulus, this process should only rely on EVC at the early, feedforward stage. Although data from both functions overlap at 80–100 ms post-stimulus onset, we do not find a discrete late (∼200 ms) time window at which TMS selectively interferes with visual awareness. Note that on the basis of these data we cannot exclude the possibility that the priming task relies on both conscious and unconscious prime perception and that we only interfered with the conscious component by EVC TMS, while leaving unconscious processing of the prime stimulus intact. Even if we did not know on a single trial basis whether the participants did or did not experience S1, we would have felt justified to conclude that if (a mixture of supra- and subliminal) priming remains completely intact, while awareness breaks down, that priming does not rely on visual awareness at this stage (otherwise we would at least expect to see a reduction, though not an abolishment, of priming).

Second, the fast, transient magnocellular visual pathway has been suggested to carry sufficient information for behavioral priming, but visual awareness does not arise until the slow, sustained parvocellular activity has reached the visual cortex [51]. Visual masking exploits the different dynamics of these two pathways, and occurs when the prime-mask SOA is chosen such that, through interchannel connections, the magnocellular (M) activity related to the mask can suppress the parvocellular (P) activity belonging to the prime stimulus [30], [49], [52]. Accordingly, EVC TMS would affect behavioral priming and visual awareness at two distinct functional time points. Indeed, we find a shift in the critical time windows of 20 ms as behavioral priming was affected by EVC TMS 60–100 ms after prime onset, and visual awareness after 80–120 ms. The transient nature of the magnocellular pathway would however predict a narrower time span of priming reduction, if only driven by depression of magnocellular activity. In a recent study on masked priming with stimuli of varied contrast, Tapia and Breitmeyer [53] showed that the size of the priming effect resembled the contrast-response function of M-neurons during conscious vision, and the contrast-response function of P-neurons during unconscious vision. Based on these results, the authors put forward a model that assigns the magnocellular pathway a critical role in conscious vision: it projects to the dorsal visual processing stream and pre-frontal cortex, which in turn potentiate the stimulus-related recurrent activity instantiated in the ventral visual stream by the activation of the parvocellular channel. They propose that the feedforward activity in the parvocellular pathway and cortical ventral stream pathways might cause the behavioral priming effect. This would mean that visual awareness and priming both rely on processing in the parvocellular stream. The model would concretely predict that the functional relevance of EVC for behavioral priming and visual awareness overlap, at the moment when EVC receives the slow parvocellular input. Moreover, the temporal window at which EVC TMS is detrimental should be broader in the case of visual awareness, because this process depends on both the early input of the faster magnocellular channel, and on the late recurrent activity in the ventral visual stream.

Our results indicate that EVC is indeed functionally relevant for both priming and visual awareness at overlapping post-stimulus time points. However, this interval is not broader for visual awareness compared to priming, and we did not find a second, discrete time period at which only visual awareness is hindered. On the other hand, we found the time period of TMS-induced impairment to be shifted 20 ms forward for subjective awareness, running from 80 to 120 ms instead of the 60 to 100 ms post-stimulus onset at which priming is impaired. These data seem to best fit the model of Koivisto et al. (2011) that considers the last phase of the subjective awareness effect, when task performance is no longer hindered by EVC TMS, as representative of local recurrent activity responsible for the generation of visual awareness [34], [54]. The idea that recurrent processing in EVC is a precondition for visual awareness has been raised before [33], [35], [55], [56], but it was suggested to consist of backprojections from distant cortical areas [57] feeding the information back to EVC at a later time point, namely around 200 ms post-stimulus [3]. As we do not find a second post-stimulus time window of TMS-induced masking around this latency, but we do seem to find a dissociation between behavioral priming and visual awareness for the 120 ms post-stimulus TMS time window, our data seem to render support for local recurrent activity as a prerequisite for visual awareness. Prime awareness is not required for behavioral priming, so the impairment is not hindered by EVC TMS around 120 ms, when this interferes with local recurrent processes. Nevertheless, this still does not explain why TMS already affects behavioral priming 60 ms after prime onset, a time point at which EVC apparently does not play a role for visual awareness yet.

### Pre-stimulus effects of EVC TMS on visual perception

The second time period of successful TMS-induced masking occurred when the magnetic pulse *preceded* the visual stimulus by 40 to 80 ms. This pre-stimulus TMS effect is much less established, and though it has been reported in previous studies [2], [4], [6], [9], [17], [18], [39], it remains controversial. Its neural origin has particularly been called into question [2], [4], [39]. Recently, we have provided evidence against non-neural accounts by showing that eye blinking, multisensory enhancement, or heightened levels of attention do not suffice to explain the pre-stimulus TMS effect [17], [18], and we thus concluded that the pre-stimulus TMS effect likely has a neural basis in early visual cortex.

The skepticism regarding the neural nature of pre-stimulus TMS was probably inspired by the fact that a TMS pulse hindering the perception of a not-yet-presented stimulus is very unintuitive. In fact, immediate signal suppression, the proposed post-stimulus TMS mechanism of action [38], cannot underlie pre-stimulus TMS effects, since there is no cortical signal to suppress yet. However, because alpha power has been shown to correlate negatively with visual awareness [58], [59], [60], [61], and the phase of ongoing alpha oscillations was shown to predict whether or not a visual stimulus reaches awareness, we have proposed that a pre-stimulus TMS pulse might evoke neuronal activity in the alpha frequency band (∼10 Hz), that is unfavorable to later visual input [18]. The absence of any suppressive TMS effects in the vertex or sham data for the pre- and post-stimulus domains provide further evidence for the notion that both the pre- and post-stimulus TMS-induced masking effect reflect neural mechanisms in EVC (see also [17], [18]).

At first glance, a pre-stimulus dissociation between awareness and behavioral priming seemed to be revealed, in the sense that pre-stimulus TMS selectively interfered with visual awareness and objective discrimination at −60 ms prior to visual stimulus onset, whereas behavioral priming remained unaffected. The current data seemed to indicate that this behavioral pattern can be achieved by TMS-induced masking. Concretely, this would mean that the processing routes of priming and visual awareness could be made to diverge by interfering with EVC prior to prime onset. Possibly, the brain state evoked by pre-stimulus TMS still allowed some sort of shallow processing of the stimulus, which might suffice for priming. Visual awareness might require a deeper form of processing, and would therefore be more susceptible to the dominant, disturbing influence of the evoked brain state. The sham control data fitted nicely into this line of reasoning, because they did not show any effects of TMS time window, and we could thus conclude that the revealed pre-stimulus dissociation is not linked to the auditory stimulation that comes with the TMS pulse. However, after controlling for the non-neural TMS effects of sensorimotor stimulation using vertex TMS, the priming data of the pre-stimulus time windows showed to be systematically affected in terms of a vertex TMS-induced increase of the priming effect. This non-neural TMS enhancement of priming is mixed with its neural EVC TMS suppression effect leading to an absence of priming decrease in time window −80 and −60 ms. Because the TMS effects in the experimental data were composed of a non-neural and a neural process counteracting each other, we estimated the size of the neural component by subtracting the vertex data for visualization purposes in Figure 5. This revealed decreased behavioral priming in the −80 and −60 ms pre-stimulus TMS time windows, hence, the same time windows at which visual awareness is impaired. In other words, after taking the vertex data into consideration, we conclude that both visual awareness and behavioral priming rely on intact EVC at roughly the same time periods, both pre- and post-stimulus.

### Methodological considerations

Aside from these conceptual implications of our findings, we would like to also stress an interesting and remarkable methodological outcome of our study: as reported in the current study, we revealed that TMS over vertex resulted in a time-specific increase, i.e. enhancement, of the priming effect of S1 on S2. Interestingly, this baseline increase due to pre-stimulus vertex TMS proved to be task-specific. This means, while vertex TMS time-specifically enhanced the priming effect of S1 on S2, it did not affect recognition accuracy or subjective awareness of S1. The lack of any effect on recognition accuracy at any of the tested vertex TMS time windows cannot be ascribed to a ceiling effect, because baseline accuracy was not perfect (i.e. ∼90% correct on average), leaving room for potential improvement.

We suggest that the susceptibility of the tasks to attentional modulation might differ. In the priming task, participants were explicitly instructed to pay attention to, and respond to, S2. Because S1 did not carry any task-relevant information (50% of trials were congruent, 50% were incongruent), we can safely assume that they did not direct attention towards S1 in any top-down fashion. Any cue drawing bottom-up attention to S1, such as the TMS pulse, could therefore have a significant beneficial influence on stimulus processing. The elevated level of attention in the early TMS time windows due to the alerting TMS pulse indeed caused a baseline increase in priming, reflecting a bigger behavioral impact of S1 on S2. In the recognition task participants were already attentive towards S1, since they were required to respond to it. Thus, less gain was to be expected from the non-neural alerting aspects of the TMS pulse, and, in accordance with this expectation, accuracy did not improve.

Sham TMS does not have the attention drawing effect that we see in the vertex data, which asks for a comparison of the two types of TMS control. Both sham and vertex TMS share the clicking sound generated in the (placebo) coil. We can rule out the auditory stimulation per se as the source of the pre-stimulus alerting effect, because the effect does not show in the sham TMS data. Sham TMS and vertex data differ when it comes to the sensory stimulation of the scalp, which is present in real TMS, and therefore also in control site TMS, but which is absent in the case of sham stimulation. The mildly aversive somatosensory experience, rather than the auditory experience, appears to be responsible for the alerting effect present in the pre-stimulus vertex data.

Generalizing, we can say that if the task under investigation requires little top-down attention, the alerting influence of skull sensations accompanying pre-stimulus (or even post-stimulus TMS) is higher, and consequently, extra care should be taken when choosing the appropriate control conditions.

## Supporting Information

Figure S1 **Individual data for the recognition task.** Average percentage correct responses (red line) and average percentage ‘seen’ stimuli (blue line) per TMS time window. Left column represents data of the experimental EVC TMS condition. Middle column represents data of the Vertex TMS control condition. Right column represents data of the Sham TMS control condition.
(TIF)

Figure S2 **Individual data for the behavioral priming task.** Average priming effect (PE), defined as the reaction times (RTs) in milliseconds on incongruent trials minus the RTs on congruent trials per TMS time window. Left column represents data of the experimental EVC TMS condition. Middle column represents data of the Vertex TMS control condition. Right column represents data of the Sham TMS control condition.
(TIF)

## References

[1] Amassian VE, Cracco RQ, Maccabee PJ, Cracco JB, Rudell A, et al (1989) Suppression of visual perception by magnetic coil stimulation of human occipital cortex. Electroencephalogr Clin Neurophysiol 74: 458–462. 2480226 10.1016/0168-5597(89)90036-1

[2] Beckers G, Homberg V (1991) Impairment of visual perception and visual short term memory scanning by transcranial magnetic stimulation of occipital cortex. Exp Brain Res 87: 421–432. 1769392 10.1007/BF00231859

[3] Camprodon JA, Zohary E, Brodbeck V, Pascual-Leone A (2009) Two phases of V1 activity for visual recognition of natural images. J Cogn Neurosci 22: 1262–1269. 10.1162/jocn.2009.21253 PMC3369215 19413482

[4] Corthout E, Uttl B, Juan CH, Hallett M, Cowey A (2000) Suppression of vision by transcranial magnetic stimulation: a third mechanism. Neuroreport 11: 2345–2349. 10943683 10.1097/00001756-200008030-00003

[5] Corthout E, Uttl B, Walsh V, Hallett M, Cowey A (1999) Timing of activity in early visual cortex as revealed by transcranial magnetic stimulation. Neuroreport 10: 2631–2634. 10574382 10.1097/00001756-199908200-00035

[6] Corthout E, Uttl B, Ziemann U, Cowey A, Hallett M (1999) Two periods of processing in the (circum)striate visual cortex as revealed by transcranial magnetic stimulation. Neuropsychologia 37: 137–145. 10080371 10.1016/s0028-3932(98)00088-8

[7] Jolij J, Lamme VA (2005) Repression of unconscious information by conscious processing: evidence from affective blindsight induced by transcranial magnetic stimulation. Proc Natl Acad Sci U S A 102: 10747–10751. 16030150 10.1073/pnas.0500834102 PMC1180757

[8] Lamme VA (2006) Zap! Magnetic tricks on conscious and unconscious vision. Trends Cogn Sci 10: 193–195. 16584910 10.1016/j.tics.2006.03.002

[9] Laycock R, Crewther DP, Fitzgerald PB, Crewther SG (2007) Evidence for fast signals and later processing in human V1/V2 and V5/MT+: A TMS study of motion perception. J Neurophysiol 98: 1253–1262. 17634339 10.1152/jn.00416.2007

[10] Ro T (2008) Unconscious vision in action. Neuropsychologia 46: 379–383. 17949759 10.1016/j.neuropsychologia.2007.09.005

[11] Sack AT, van der Mark S, Schuhmann T, Schwarzbach J, Goebel R (2009) Symbolic action priming relies on intact neural transmission along the retino-geniculo-striate pathway. Neuroimage 44: 284–293. 18721890 10.1016/j.neuroimage.2008.07.030

[12] de Graaf TA, Herring J, Sack AT (2011) A chronometric exploration of high-resolution ‘sensitive TMS masking’ effects on subjective and objective measures of vision. Exp Brain Res 209: 19–27. 21161191 10.1007/s00221-010-2512-z PMC3035793

[13] Koivisto M, Mantyla T, Silvanto J (2010) The role of early visual cortex (V1/V2) in conscious and unconscious visual perception. Neuroimage 51: 828–834. 20188199 10.1016/j.neuroimage.2010.02.042

[14] Overgaard M, Nielsen JF, Fuglsang-Frederiksen A (2004) A TMS study of the ventral projections from V1 with implications for the finding of neural correlates of consciousness. Brain Cogn 54: 58–64. 14733901 10.1016/s0278-2626(03)00260-4

[15] de Graaf TA, Goebel R, Sack AT (2011) Feedforward and quick recurrent processes in early visual cortex revealed by TMS? Neuroimage 61: 651–659. 22032946 10.1016/j.neuroimage.2011.10.020

[16] Koivisto M, Railo H, Revonsuo A, Vanni S, Salminen-Vaparanta N (2011) Recurrent processing in V1/V2 contributes to categorization of natural scenes. J Neurosci 31: 2488–2492. 21325516 10.1523/JNEUROSCI.3074-10.2011 PMC6623680

[17] de Graaf TA, Cornelsen S, Jacobs C, Sack AT (2011) TMS effects on subjective and objective measures of vision: stimulation intensity and pre- versus post-stimulus masking. Conscious Cogn 20: 1244–1255. 21632262 10.1016/j.concog.2011.04.012

[18] Jacobs C, Goebel R, Sack AT (2012) Visual awareness suppression by pre-stimulus brain stimulation; a neural effect. Neuroimage 59: 616–624. 21840406 10.1016/j.neuroimage.2011.07.090

[19] Weiskrantz L (2009) Blindsight: a case study spanning 35 years and new developments. Oxford Oxford University Press.

[20] Lau HC, Passingham RE (2006) Relative blindsight in normal observers and the neural correlate of visual consciousness. Proc Natl Acad Sci U S A 103: 18763–18768. 17124173 10.1073/pnas.0607716103 PMC1693736

[21] Breitmeyer BG, Ogmen H, Chen J (2004) Unconscious priming by color and form: different processes and levels. Conscious Cogn 13: 138–157. 14990249 10.1016/j.concog.2003.07.004

[22] Ro T, Singhal NS, Breitmeyer BG, Garcia JO (2009) Unconscious processing of color and form in metacontrast masking. Atten Percept Psychophys 71: 95–103. 19304600 10.3758/APP.71.1.95

[23] Vorberg D, Mattler U, Heinecke A, Schmidt T, Schwarzbach J (2003) Different time courses for visual perception and action priming. Proc Natl Acad Sci U S A 100: 6275–6280. 12719543 10.1073/pnas.0931489100 PMC156362

[24] Kouider S, Dehaene S (2009) Subliminal number priming within and across the visual and auditory modalities. Exp Psychol 56: 418–433. 19502203 10.1027/1618-3169.56.6.418

[25] Kouider S, Dehaene S, Jobert A, Le Bihan D (2007) Cerebral bases of subliminal and supraliminal priming during reading. Cereb Cortex 17: 2019–2029. 17101688 10.1093/cercor/bhl110

[26] Van den Bussche E, Notebaert K, Reynvoet B (2009) Masked primes can be genuinely semantically processed: a picture prime study. Exp Psychol 56: 295–300. 19447745 10.1027/1618-3169.56.5.295

[27] van Gaal S, Lamme VA, Ridderinkhof KR (2010) Unconsciously triggered conflict adaptation. PLoS One 5: e11508. 20634898 10.1371/journal.pone.0011508 PMC2901348

[28] Lau HC, Passingham RE (2007) Unconscious activation of the cognitive control system in the human prefrontal cortex. The Journal of neuroscience : the official journal of the Society for Neuroscience 27: 5805–5811. 17522324 10.1523/JNEUROSCI.4335-06.2007 PMC6672767

[29] Koivisto M, Henriksson L, Revonsuo A, Railo H (2012) Unconscious response priming by shape depends on geniculostriate visual projection. Eur J Neurosci 35: 623–633. 22304409 10.1111/j.1460-9568.2011.07973.x

[30] Breitmeyer BG, Ro T, Ogmen H (2004) A comparison of masking by visual and transcranial magnetic stimulation: implications for the study of conscious and unconscious visual processing. Conscious Cogn 13: 829–843. 15522634 10.1016/j.concog.2004.08.007

[31] Koivisto M, Henriksson L, Revonsuo A, Railo H (2012) Unconscious response priming by shape depends on geniculostriate visual projection. Eur J Neurosci 35: 623–633. 22304409 10.1111/j.1460-9568.2011.07973.x

[32] Heinen K, Jolij J, Lamme VA (2005) Figure-ground segregation requires two distinct periods of activity in V1: a transcranial magnetic stimulation study. Neuroreport 16: 1483–1487. 16110276 10.1097/01.wnr.0000175611.26485.c8

[33] Bullier J (2001) Feedback connections and conscious vision. Trends Cogn Sci 5: 369–370. 11520692 10.1016/s1364-6613(00)01730-7

[34] Koivisto M, Railo H, Salminen-Vaparanta N (2011) Transcranial magnetic stimulation of early visual cortex interferes with subjective visual awareness and objective forced-choice performance. Conscious Cogn 20: 288–298. 20863717 10.1016/j.concog.2010.09.001

[35] Lamme VA, Roelfsema PR (2000) The distinct modes of vision offered by feedforward and recurrent processing. Trends Neurosci 23: 571–579. 11074267 10.1016/s0166-2236(00)01657-x

[36] Pascual-Leone A, Walsh V (2001) Fast backprojections from the motion to the primary visual area necessary for visual awareness. Science 292: 510–512. 11313497 10.1126/science.1057099

[37] Fahrenfort JJ, Scholte HS, Lamme VA (2007) Masking disrupts reentrant processing in human visual cortex. J Cogn Neurosci 19: 1488–1497. 17714010 10.1162/jocn.2007.19.9.1488

[38] Harris JA, Clifford CW, Miniussi C (2008) The functional effect of transcranial magnetic stimulation: signal suppression or neural noise generation? J Cogn Neurosci 20: 734–740. 18052790 10.1162/jocn.2008.20048

[39] Sack AT, Kohler A, Linden DE, Goebel R, Muckli L (2006) The temporal characteristics of motion processing in hMT/V5+: combining fMRI and neuronavigated TMS. Neuroimage 29: 1326–1335. 16185899 10.1016/j.neuroimage.2005.08.027

[40] Amassian VE, Cracco RQ, Maccabee PJ, Cracco JB, Rudell AP, et al (1998) Transcranial magnetic stimulation in study of the visual pathway. Journal of clinical neurophysiology : official publication of the American Electroencephalographic Society 15: 288–304. 9736464 10.1097/00004691-199807000-00002

[41] Thielscher A, Reichenbach A, Ugurbil K, Uludag K (2010) The cortical site of visual suppression by transcranial magnetic stimulation. Cereb Cortex 20: 328–338. 19465739 10.1093/cercor/bhp102 PMC4810000

[42] Salminen-Vaparanta N, Noreika V, Revonsuo A, Koivisto M, Vanni S (2012) Is selective primary visual cortex stimulation achievable with TMS? Human brain mapping 33: 652–665. 21416561 10.1002/hbm.21237 PMC6870472

[43] de Graaf TA, Sack AT (2011) Null results in TMS: from absence of evidence to evidence of absence. Neurosci Biobehav Rev 35: 871–877. 20955732 10.1016/j.neubiorev.2010.10.006

[44] Hackley SA (2009) The speeding of voluntary reaction by a warning signal. Psychophysiology 46: 225–233. 18811626 10.1111/j.1469-8986.2008.00716.x

[45] Skotte JH, Nojgaard JK, Jorgensen LV, Christensen KB, Sjogaard G (2007) Eye blink frequency during different computer tasks quantified by electrooculography. Eur J Appl Physiol 99: 113–119. 17115181 10.1007/s00421-006-0322-6

[46] Boyer JL, Harrison S, Ro T (2005) Unconscious processing of orientation and color without primary visual cortex. Proc Natl Acad Sci U S A 102: 16875–16879. 16263934 10.1073/pnas.0505332102 PMC1283801

[47] Rahnev DA, Maniscalco B, Luber B, Lau H, Lisanby SH (2012) Direct injection of noise to the visual cortex decreases accuracy but increases decision confidence. Journal of neurophysiology 107: 1556–1563. 22170965 10.1152/jn.00985.2011

[48] Eimer M, Schlaghecken F (2003) Response facilitation and inhibition in subliminal priming. Biol Psychol 64: 7–26. 14602353 10.1016/s0301-0511(03)00100-5

[49] Ogmen H, Breitmeyer BG, Melvin R (2003) The what and where in visual masking. Vision Res 43: 1337–1350. 12742104 10.1016/s0042-6989(03)00138-x

[50] Schacter DL, Buckner RL (1998) Priming and the brain. Neuron 20: 185–195. 9491981 10.1016/s0896-6273(00)80448-1

[51] Paulus W, Korinth S, Wischer S, Tergau F (1999) Differential inhibition of chromatic and achromatic perception by transcranial magnetic stimulation of the human visual cortex. Neuroreport 10: 1245–1248. 10363933 10.1097/00001756-199904260-00017

[52] Breitmeyer BG, Ogmen H (2000) Recent models and findings in visual backward masking: a comparison, review, and update. Perception & psychophysics 62: 1572–1595. 11140180 10.3758/bf03212157

[53] Tapia E, Breitmeyer BG (2011) Visual consciousness revisited: magnocellular and parvocellular contributions to conscious and nonconscious vision. Psychol Sci 22: 934–942. 21697525 10.1177/0956797611413471

[54] Koivisto M, Silvanto J (2011) Relationship between visual binding, reentry and awareness. Conscious Cogn 20: 1293–1303. 21398146 10.1016/j.concog.2011.02.008

[55] Lamme VA (2001) Blindsight: the role of feedforward and feedback corticocortical connections. Acta Psychol (Amst) 107: 209–228. 11388136 10.1016/s0001-6918(01)00020-8

[56] Tong F (2003) Primary visual cortex and visual awareness. Nat Rev Neurosci 4: 219–229. 12612634 10.1038/nrn1055

[57] Lamme VA (2006) Towards a true neural stance on consciousness. Trends Cogn Sci 10: 494–501. 16997611 10.1016/j.tics.2006.09.001

[58] Thut G, Nietzel A, Brandt SA, Pascual-Leone A (2006) Alpha-band electroencephalographic activity over occipital cortex indexes visuospatial attention bias and predicts visual target detection. J Neurosci 26: 9494–9502. 16971533 10.1523/JNEUROSCI.0875-06.2006 PMC6674607

[59] Toscani M, Marzi T, Righi S, Viggiano MP, Baldassi S (2010) Alpha waves: a neural signature of visual suppression. Exp Brain Res 207: 213–219. 20972777 10.1007/s00221-010-2444-7

[60] van Dijk H, Schoffelen JM, Oostenveld R, Jensen O (2008) Prestimulus oscillatory activity in the alpha band predicts visual discrimination ability. J Neurosci 28: 1816–1823. 18287498 10.1523/JNEUROSCI.1853-07.2008 PMC6671447

[61] Mathewson KE, Gratton G, Fabiani M, Beck DM, Ro T (2009) To see or not to see: prestimulus alpha phase predicts visual awareness. J Neurosci 29: 2725–2732. 19261866 10.1523/JNEUROSCI.3963-08.2009 PMC2724892

