# Supplementary figures and images for: The Temporal Dynamics of Early Visual Cortex Involvement in Behavioral Priming
Source: PLoS One. 2012 Nov 14;7(11):e48808. doi: 10.1371/journal.pone.0048808 (PMC3498241; doi:10.1371/journal.pone.0048808)

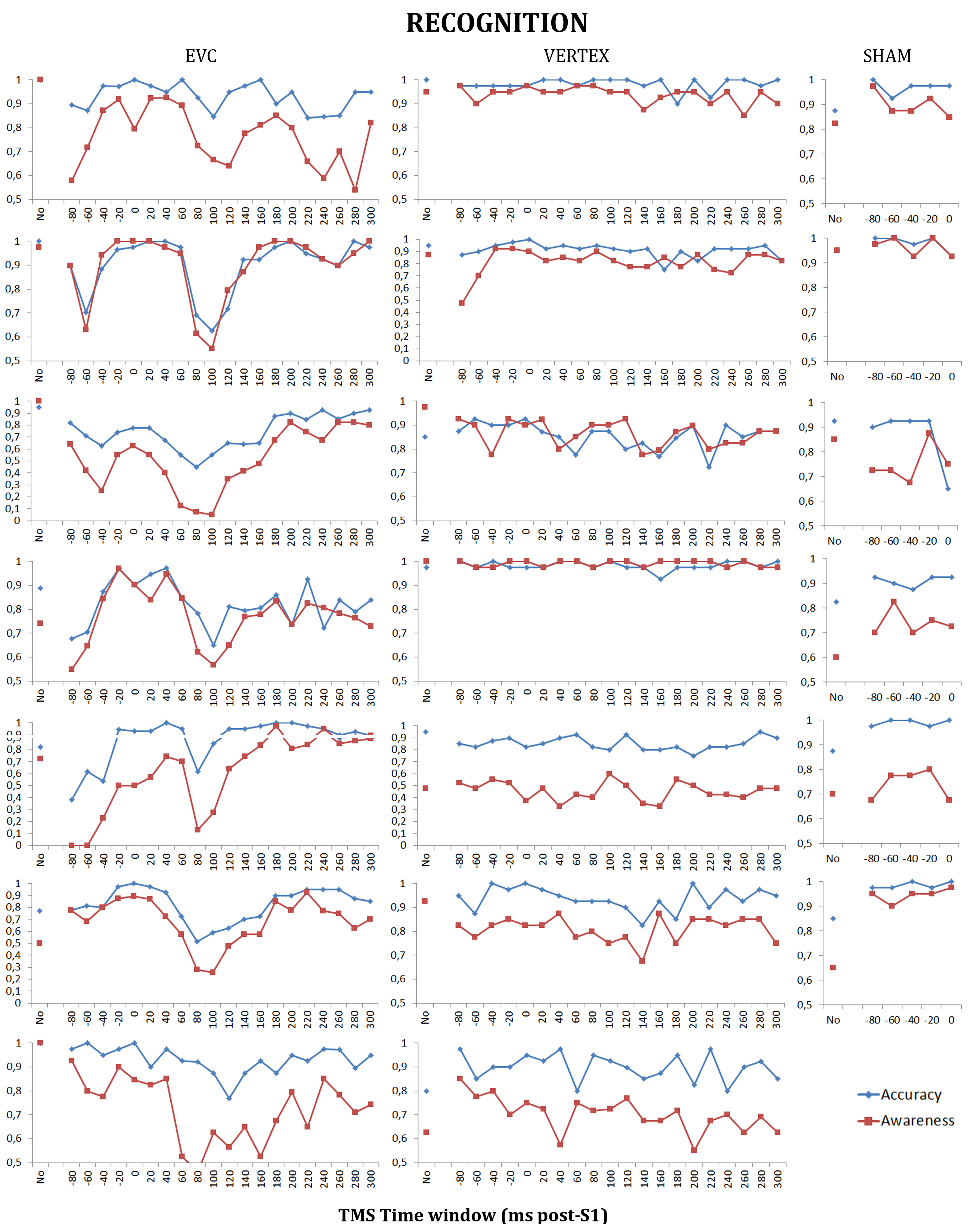

Supplement: Figure S1 — Individual data for the recognition task. Average percentage correct responses (red line) and average percentage ‘seen’ stimuli (blue line) per TMS time window. Left column represents data of the experimental EVC TMS condition. Middle column represents data of the Vertex TMS control condition. Right column represents data of the Sham TMS control condition. (TIF) [file pone.0048808.s001.tif]

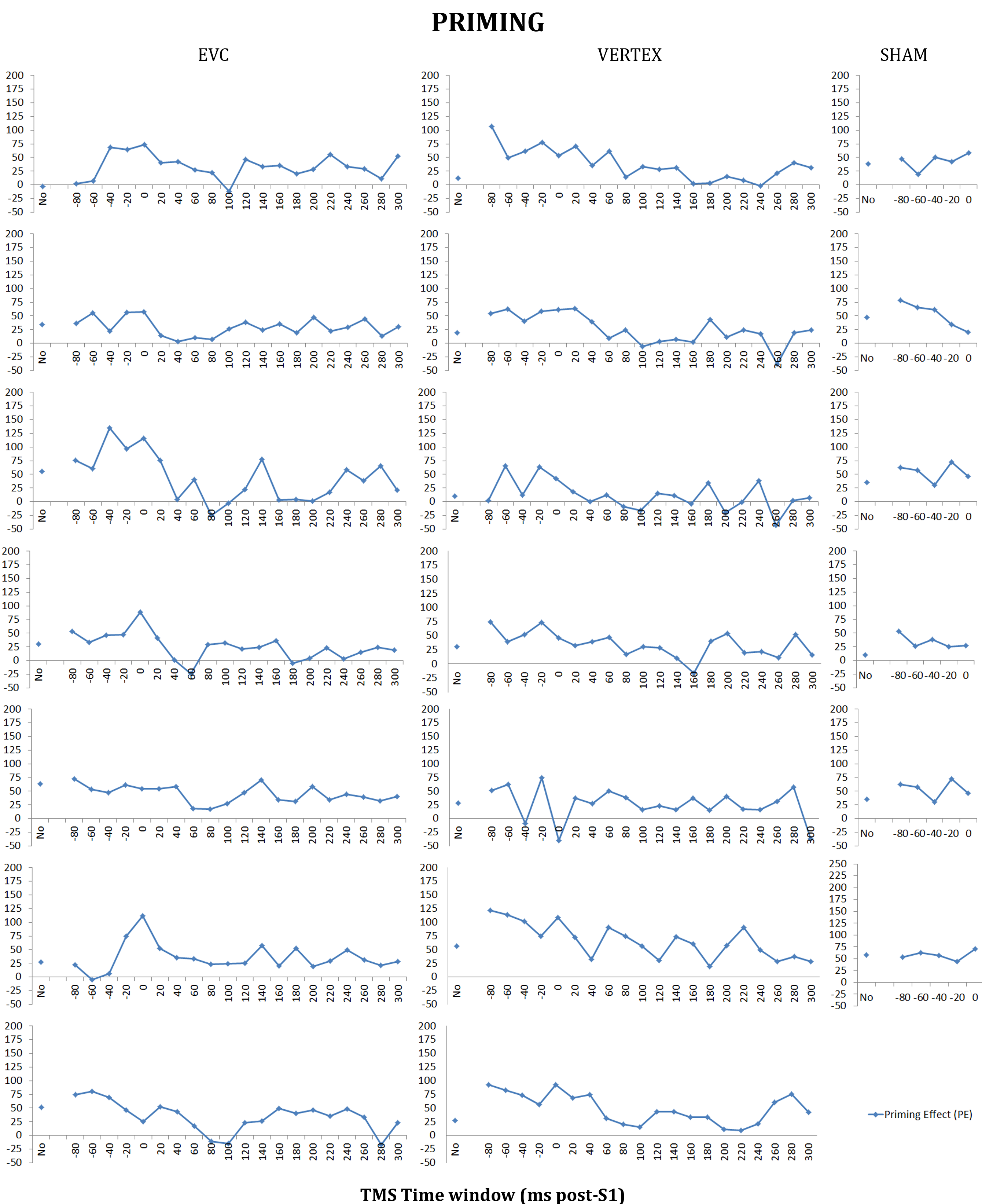

Supplement: Figure S2 — Individual data for the behavioral priming task. Average priming effect (PE), defined as the reaction times (RTs) in milliseconds on incongruent trials minus the RTs on congruent trials per TMS time window. Left column represents data of the experimental EVC TMS condition. Middle column represents data of the Vertex TMS control condition. Right column represents data of the Sham TMS control condition. (TIF) [file pone.0048808.s002.tif]
